# The pathology informatics curriculum wiki: Harnessing the power of user-generated content

**DOI:** 10.4103/2153-3539.65428

**Published:** 2010-07-13

**Authors:** Ji Yeon Kim, Thomas M. Gudewicz, Anand S. Dighe, John R. Gilbertson

**Affiliations:** Department of Pathology, Massachusetts General Hospital and Harvard Medical School, Boston, MA, USA

**Keywords:** Wikipedia, Wiki, On-line Education, Pathology Informatics Curriculum

## Abstract

**Background:**

The need for informatics training as part of pathology training has never been so critical, but pathology informatics is a wide and complex field and very few programs currently have the resources to provide comprehensive educational pathology informatics experiences to their residents. In this article, we present the “pathology informatics curriculum wiki”, an open, on-line wiki that indexes the pathology informatics content in a larger public wiki, Wikipedia, (and other online content) and organizes it into educational modules based on the 2003 standard curriculum approved by the Association for Pathology Informatics (API).

**Methods and Results:**

In addition to implementing the curriculum wiki at http://pathinformatics.wikispaces.com, we have evaluated pathology informatics content in Wikipedia. Of the 199 non-duplicate terms in the API curriculum, 90% have at least one associated Wikipedia article. Furthermore, evaluation of articles on a five-point Likert scale showed high scores for comprehensiveness (4.05), quality (4.08), currency (4.18), and utility for the beginner (3.85) and advanced (3.93) learners. These results are compelling and support the thesis that Wikipedia articles can be used as the foundation for a basic curriculum in pathology informatics.

**Conclusions:**

The pathology informatics community now has the infrastructure needed to collaboratively and openly create, maintain and distribute the pathology informatics content worldwide (Wikipedia) and also the environment (the curriculum wiki) to draw upon its own resources to index and organize this content as a sustainable basic pathology informatics educational resource. The remaining challenges are numerous, but largest by far will be to convince the pathologists to take the time and effort required to build pathology informatics content in Wikipedia and to index and organize this content for education in the curriculum wiki.

## INTRODUCTION

### The Need for Informatics in Pathology

Information from medical laboratories and pathologists is critical to modern medicine across the entire continuum of patient care. The laboratory provides consultative services and information management in the form of reliable, reproducible, largely quantitative data that are used extensively to screen populations, diagnose disease, estimate prognosis, guide therapy and measure outcomes.[1] The importance of the laboratory as an information source is seen in a study which demonstrated tht 94% of requests to a clinical electronic medical record were for laboratory results alone.[2] It is believed that more than half of the significant medical decisions are based, at least partially, on information from the laboratory.[3,4]

The increasingly data-intensive nature of medical practice, the critical role of pathology data to this practice, and the growing use of information systems to manage and communicate information caused visionary pathologists like Korpman[5] and Friedman[6] to recommend training in pathology informatics as an important component of preparing pathology residents to be become the “medical information specialist.”

Early definitions of pathology informatics, such as that by Friedman in his seminal 1990 paper,[6] focused on information management, particularly, the communication and processing of laboratory data, and emphasized the strategic importance of these activities to pathology practice. Today, these activities are becoming even more important with the rise of evidence-based medicine, personalized medicine, public demand for improved quality and patient safety in healthcare, and use of electronic health records.[7–9]

Furthermore, the creation and processing of pathology data is changing. Laboratories are increasingly automated and are managed through data-driven management protocols such as Six Sigma[10–12] and LEAN.[13–15] Even anatomic pathology is becoming digitized,[16–20] and computer analysis and decision support is becoming more common.[21–25] Pathologists are commonly asked to manage complex, spatially distributed facilities (and point of care networks), generate standardized and coded “machine consumable” data, create “integrated” reports from data generated by multiple tests in multiple labs and communicate with multiple hospital systems. Informatics now covers the total testing cycle, from ordering to transporting, processing, testing, resulting, interpreting, integrating, communicating, advising and documenting.[26–28] The importance of understanding informatics and the need for informatics training as part of pathology training has never been so critical and will become increasingly important with the growing dependence on medical information systems.

### The Availability of Pathology Informatics Training

Between 1993 and 2003, repeated surveys of pathology residency programs in the United States and Canada have shown that between 19 and 26% of residency programs offered “dedicated rotations” in pathology informatics, with the reported percentage actually decreasing in that time period[29–31] [Table 1]. The surveys also demonstrated significant variability in the scope and quality of pathology informatics training. Reported methods of teaching included computer training courses to gain proficiency with laboratory information system (LIS) applications, lecture series (from less than 1 week to periodic series spanning years), direct use (hands-on) with computers, and self-study, with most programs using more than one instructional method. A concern raised by the authors of the surveys is that many pathology residency programs view training in informatics as equivalent to use of the LIS and perhaps use of personal computers.[29–31]

**Table 1:** Surveys of US and Canadian pathology residency programs

Published survey – year published	% (n) offering a dedicated informatics rotation
Balis, Aller, Ashwood – 1993	26 (37/142)
Goldberg-Kahn and Healy – 1997	24 (20/84)
Henricks and Healy – 2002	19 (13/72)

Proposals for a standardized curriculum for pathology informatics training in residencies began as early as 1992 with recommendations by Peters and Clark[32] and Buffone and Beck.[33] In 2003, a proposal for a standardized basic pathology informatics curriculum, based largely on the publication by Henricks, Boyer, Harrison, Tuthill and Healy,[34] was supported by the Training and Education Committee of the Association for Pathology Informatics (API).

In 2000, the University of Pittsburgh Department of Pathology (Pittsburgh, PA, USA) established a 3-week full-time core rotation in pathology informatics[35,36] which incorporated many of the curriculum concepts proposed in the articles cited above. It included an introduction to general desktop computing applications and the use of the internet, as well as vocabularies, ontologies, imaging, interfaces, databases and laboratory information systems. The rotation included 63 hours of didactic sessions and hands-on laboratories, was taught by 19 faculty and staff (including one of the authors of this article, JRG) and was required for residents at the beginning of their second year of training.

One of the major lessons of the early Pittsburgh experience was that extensive resources and planning were required to implement a comprehensive pathology informatics curriculum in a residency program. There were far more faculty than students, and most faculty members gave more than one presentation. Only a small number of programs have expertise and resources to present such an educational experience. For example, according to the API, there are only five US medical centers (including the University of Pittsburgh) that support pathology informatics fellowships[37] [Table 2]. Thus, considerations of resources and expertise seem to indicate that the comprehensive teaching of informatics remains limited to a few specialized academic centers.

**Table 2:** Pathology informatics fellowships in the USA

Programs offering a pathology informatics fellowship (in alphabetical order)	Number of fellows	Duration of training (years; default is 1 if not specified)
Henry Ford Hospital and Health System	2	2
Johns Hopkins Hospital	Not specified	1–2
Partners HealthCare (Massachusetts General	2–3	1–2
Hospital, Brigham and Women's Hospital and
Northshore Medical Center)
University of Michigan	1	1
University of Pittsburgh	1–2	1

Another important lesson from the early University of Pittsburgh experience is the problem of sustainability. Finding 19 faculty and staff willing to dedicate 3 weeks to teach a small number of residents every year was difficult, even at a large institution such as Pittsburgh. Presently, the University of Pittsburgh still runs an outstanding course, but it is smaller and shorter than it was in 2003. A compounding burden is that a pathology informatics curriculum needs to keep pace with rapid changes in technology, standards and practices. For example, terms like “floppy disk” from the standardized curriculum of 2003 (*vide* supra) come across as dated, and possibly irrelevant, only a few years later. Any proposed standard training program in pathology informatics must consider that the current academic pathology informatics community is still small and the effort necessary to create and update any curriculum is substantial.

Partially in response to the issues above, the University of Pittsburgh recently created the Virtual Rotation in Pathology Informatics (VRPI).[38] Based on the initial proposals for a standard curriculum (*vide* supra), this extension of the original Pittsburgh pathology informatics rotation is available online at https://secure.opi.upmc.edu/VRPI/index.cfm. The rotation includes original lectures, video-recorded laboratory exercises and reading assignments from the textbook “Practical Pathology Informatics” by John Sinard.[39] Significantly, it also leverages other educational resources on the internet, including lectures in the online archives of the APIII conference (Advancing Practice, Instruction and Innovation through Informatics; http://apiii.upmc.edu/), and tutorials from the National Center for Biotechnology Information (NCBI) and PubMed.

### Wikis and the Power of User Generated Content

Originally, the internet was a technology for the efficient distribution of electronic content, making it an appropriate vehicle for online curricula and web-based learning. The internet makes educational material accessible to anyone with an internet connection for “anywhere, anytime” learning. Such capabilities can reduce the need for local expertise. Because of this convenience, the internet is used by an estimated 40 million adults in the US (20% of Americans) as their primary source of news and information about science, second only to television, according to a report by the Pew Internet and American Life Project.[40] In addition, the same study found that the internet is a research tool for 87% of online users (about 128 million adults) and it is the first source people would turn to if they had a question on a specific scientific topic. The internet can provide more widespread accessibility to pathology informatics training materials, but the creation and maintenance of these materials in a rapidly changing, technology-heavy field like pathology informatics, with a limited number of academic experts (with limited cycles for teaching), is still problematic.

More recently, “Web 2.0” technologies are changing the internet from a distribution publication medium to a place for distributed *creation* and reuse of content. The term Web 2.0 became popular following the O'Reilly Media Web 2.0 Conference in 2004, and refers to collaborative information-sharing platforms that include wikis, blogs, and podcasts.[41] The wiki is a particularly interesting construct in the context of creating and sharing a pathology informatics curriculum. Wiki software (from the Hawaiian *wiki*, to hurry, swift) allows multiple users to easily collaborate as coauthors to create, distribute, edit and update content. Initially designed in 1994, it allows creation and editing of interlinked web pages via a web browser using a simplified mark-up language, or a WYSIWYG (“What You See Is What You Get”) text editor.[42]

The use of wikis and other Web 2.0 technologies in medical education and the health sciences has been widely discussed in the literature,[43–55] with 45% of medical schools and 53% of nursing schools in a recent survey reporting use of blogs, wikis, videocasts, and podcasts.[56] Use of Web 2.0 tools has taken hold among health educators, clinicians, librarians and patients alike, with numerous articles, journal issues and conferences devoted to developments in this field.[57,58]

At Massachusetts General Hospital, available pathology informatics resources include 12 Harvard Medical School faculty members, each independent with individual resources, areas of expertise and points of view. Our initial idea was to use a wiki as an internal tool to build a curriculum for our residents. However, we quickly realized that with or without a wiki, we did not have enough people with enough time to create and sustain a comprehensive course. We continued to focus on the scale and scope of the project to identify alternate solutions, leading us to the concept of a wiki-based course using Wikipedia as its primary reference.

**Figure 1:**
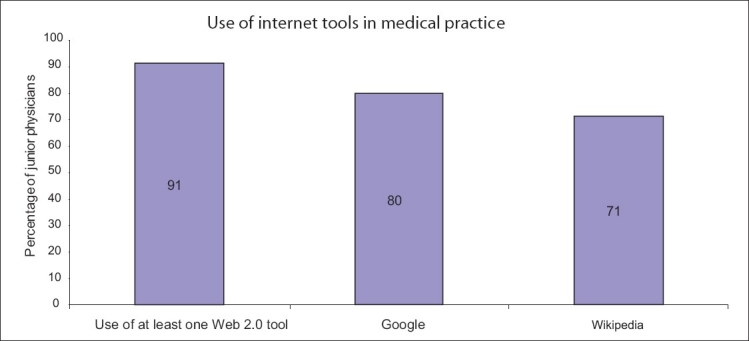
Internet and Web 2.0 play an important role in information seeking for both clinical decisions and medical education among recently trained junior physicians.[41]

Several Web traffic measuring firms report that Wikipedia (http://wikipedia.org/) is the most popular educational and reference site on the internet, and is in the top ten of the most heavily visited sites overall, including search engine sites.[59,60] As of 03/06/2010, with more than 3 million English articles and 68 million monthly visitors, Wikipedia has an estimated 91,000 active contributors collectively making 10,000 edits every 6–8 weeks.[61,62] Wikipedia is a prominent source of high-quality online health-related information, even when compared with other health references such as Medline Plus.[63] In one study, 80 and 70% of recently trained junior physicians (2–3 years post-graduation) already use Web 2.0 tools such as Google and Wikipedia, respectively, for information-seeking activities for clinical decisions and medical education[64] [Figure 1]. There is even a community dedicated to improving medicine and health-related articles of Wikipedia called WikiProject Medicine (http://en.wikipedia.org/wiki/Wikipedia:WikiProject_Medicine).

An initial evaluation of Wikipedia identified multiple potential articles for use as the core of a pathology informatics curriculum. We then asked the following questions: 1) was the pathology informatics information represented in Wikipedia articles comprehensive, of high quality, and up-to-date; 2) what would be the best way to index and present these articles in a coherent course that would be useful to students; 3) what would be the best way to maintain the index and the lessons; 4) if Wikipedia articles important to pathology informatics did not exist or were not of high quality, how could the articles be created, improved or supplemented and 5) would such an initiative be consistent with Wikipedia's rules for fair use.

Given this background, we proposed the Pathology Informatics Curriculum Wiki (http://pathinformatics.wikispaces.com), an online public wiki, which, using the 2003 API standard curriculum as a guide, would act as an organized index to pathology informatics content on a much larger public wiki, Wikipedia, as well as other stable, well-established, community-based and continually updated online sources. The objective was not to create our own index or our own content, but to leverage the knowledge and skill of the entire pathology informatics community to create a shared educational resource for pathology training programs that would supplement or inspire development of local curricula in pathology informatics.

## MATERIALS AND METHODS

### Evaluation of the Pathology Informatics Content in Wikipedia

To evaluate the scope of pathology informatics information in Wikipedia, we took 198 non-duplicative terms from the 2003 proposed standard curriculum (*vide* supra) and determined the percentage mapped to Wikipedia articles by searching each of the terms on Wikipedia (as of 03/06/2010). In our analysis, we included one curriculum section heading “Laboratory Information System” as a term, for a total of 199 terms. Where there were approximate matches (e.g. “fiber-optic cable” versus “optical fiber cable”), we included the alternative terms as a match (Appendix).

**Appendix:** 

Pathology informatics terms from the standard “2003” curriculum in Henricks *et al*,[34]	In Wikipedia as of 03/06/2010	Alternative Wikipedia name	Number revisions (02/01/2009–02/01/2010)	Number tags as of 03/07/2010
Computer basics				
Computer	Yes		134	1
Hardware	Yes		45	3
Software	Yes	Computer software	381	1
Program	Yes	Computer program	63	0
Information system	Yes		20	0
Bit	Yes		90	0
Byte (kilo-, mega-, giga-, tera-)	Yes		170	0
Analog	Yes	Analog signal	79	0
Digital	Yes	Digital, digital signal	48, 22	0, 1
File	Yes	Computer file	58	1
Directory	Yes	Folder (computing)	24	0
Folder	Yes	Folder (computing)	24	0
Standard	Yes		26	1
Protocol	Yes	Protocol (computing)	104	2
American Standard Code for Information Interchange (ASCII)	Yes		155	0
Unicode	Yes		106	1
Network	Yes	Computer network	730	1
Bandwidth	Yes	Bandwidth (computing)	62	2
Backup	Yes		60	1
Redundancy	Yes	Redundancy (information theory), data redundancy	0, 8	0
Hardware				
Central processing unit (CPU)	Yes	Central processing unit	396	1
Megahertz	Yes	Hertz	101	0
Random access memory (RAM)	Yes	Random-access memory	338	0
Read-only memory (ROM)	Yes	Read-only memory	46	0
Hard disk drive (HDD)	Yes	Hard disk drive	526	3
Redundant array of independent/inexpensive disks (RAIDs)	Yes	RAID	398	4
Compact disk read-only memory (CD-ROM)	Yes	CD-ROM	140	0
Compact disk read-write (CD-RW)	Yes	CD-RW	15	1
Digital versatile disk (DVD)	Yes	DVD	498	0
Tape drive	Yes		22	0
Floppy disk	Yes		262	1
Microfiche	Yes	Microform	23	0
Bus	Yes	Bus (computing)	50	0
Card	Yes	Expansion card	20	0
Peripheral	Yes		72	0
Microcomputer	Yes		57	4
Minicomputer	Yes		38	1
Mainframe computer	Yes		162	3
Multiuser system	Yes	Multiuser	17	0
Terminal server	Yes		23	3
“Dumb” terminal	Yes	Computer terminal	18	0
Personal digital assistant (PDA)	Yes		134	6
Software				
Operating system	Yes		772	0
Graphical user interface (GUI)	Yes		167	1
Application	Yes	Application software	108	2
Programming language	Yes		286	0
Device driver	Yes		72	1
Terminal emulator	Yes		59	2
Word processor	Yes		128	0
Spreadsheet	Yes		168	2
Presentation graphics	Yes	Presentation program	57	2
Database	Yes		100	4
Electronic mail (E-mail)	Yes		362	4
License	Yes		51	1
Open source	Yes		224	1
Computer networks				
Local area network (LAN)	Yes		153	0
Network interface card (NIC)	Yes	Network interface controller	78	0
Ethernet (fast, gigabit)	Yes		225	1
Network hub	Yes	Ethernet hub	65	1
Router	Yes		181	1
Gateway	Yes	Gateway (telecommunications)	30	0
Switch	Yes		120	1
Fiber-optic cable	Yes	Optical fiber cable	34	0
Wireless	Yes		169	1
Client/server architecture	Yes	Client-server	118	3
Network operating system	Yes		34	1
Internet protocol (IP)	Yes		99	0
Port	Yes	Computer port (hardware)	23	1
File server	Yes		25	0
Application server	Yes		65	0
Print server	Yes		13	0
Middleware	Yes		34	0
“N-tier” architecture	Yes	Multitier architecture	39	1
Component software	Yes	Component-based software engineering	54	4
Cluster	Yes	Cluster (computing)	670	0
Thin client	Yes		132	1
Wide area network (WAN)	Yes		63	1
Plain old telephone service (POTS)	Yes	Plain old telephone service	19	2
Modem	Yes		131	2
Cable modem	Yes		44	1
Integrated services digital network (ISDN)	Yes	Integrated services digital network	116	4
Digital subscriber line (DSL)	Yes	Digital subscriber line	89	1
T1	Yes	Digital Signal 1	21	2
T3	Yes	Digital Signal 3	8	0
Internet related				
Internet	Yes		289	0
Internet service provider (ISP)	Yes		88	1
Intranet	Yes		134	1
Extranet	Yes		58	0
Virtual private network (VPN)	Yes		246	1
World Wide Web	Yes		565	0
Web browser	Yes		239	0
Applet	Yes		38	1
Plug-in	Yes	Plug-in (computing)	32	1
Domain name	Yes		253	3
Uniform resource locator (URL)	Yes	Uniform resource locator	225	1
File transfer protocol (FTP)	Yes	File transfer protocol	188	1
Telnet	Yes		84	0
SSH (secure shell)	Yes	Secure shell	102	0
Hypertext	Yes		74	0
Hypertext transfer protocol (http)	Yes	Hypertext transfer protocol	249	1
Markup language and tags	Yes	Markup language	77	0
Hypertext markup language (HTML)	Yes		605	0
Extensible markup language (XML)	Yes		438	0
Portable document format (PDF) document	Yes		200	1
*Laboratory information systems^1^*	Yes	Laboratory information system		1
Order entry	Yes	LIS or computer physician order entry	39	0
Accession number	No			n/a
Maintenance table	No			n/a
Mnemonic	No	Assembly mnemonics		n/a
Worksheet	No	Not specific to LIS		n/a
Cumulative report	No			n/a
Interim report	No			n/a
Management report	No			n/a
Audit trail	Yes		6	2
Remote printing	No	Remote line printer spooling system (RLPR), Internet Printing Protocol		n/a
Line printer	Yes		20	1
Bar code	Yes	Barcode	285	0
Backup	Yes			1
Fault tolerance	Yes	Fault-tolerant design	5	1
Purge	No			n/a
Instrument interface	No			n/a
Application interface	No			n/a
Interface engine	No			n/a
Translation table	No			n/a
Admission-discharge-transfer (ADT)	No			n/a
Test area	No			n/a
Database	Yes			4
Database management system (DBMS)	Yes		206	4
Query language	Yes		11	0
Structured query language (SQL)	Yes		228	0
Open database connectivity (ODBC)	Yes		28	2
Data standards and encoding schemes				
Structured medical language	No			n/a
HL7	Yes	Health level 7	39	1
ASCII	Yes			0
American Society for Testing and Materials (ASTM)	Yes	ASTM international	35	0
LOINC	Yes		4	1
DICOM	Yes	Digital imaging and communications in medicine	76	0
Systematized nomenclature of medicine (SNOMED)	Yes	SNOMED CT	24	0
ICD-9, ICD-10 (International classification of disease)	Yes	International statistical classification of diseases and related health problems	60	1
Current procedural terminology (CPT)	Yes		7	0
System management and software development				
Application service provider (ASP)	Yes		13	1
Software licensing	Yes	Software license	60	1
Requirements analysis	Yes		83	1
Request for proposal (RFP)	Yes	Request for proposal	42	0
Scope document	Yes	Scope (project management)	20	3
Technical specifications document	Yes	Specification (technical standard)	100	0
Source code	Yes		53	1
Code escrow	Yes	Source code escrow	3	0
Software version	Yes	Software versioning	45	1
Software build	Yes		1	0
Maintenance fee	Yes		1	0
Service level agreement (SLA)	Yes		63	1
Alpha and beta software	Yes	Software release life cycle	108	2
Developmental partnership	No			n/a
System manager	Yes	Systems management	16	3
User support	Yes	Technical support	91	1
Help desk	Yes		73	1
Data analysis				
Relational database	Yes		79	1
Flat file database	Yes		20	0
Hierarchical database	Yes	Hierarchical database model	20	0
Object-oriented database	Yes	Object database	23	1
Data model	Yes		57	0
Data field	Yes		0	1
Data record	Yes	Row (database)	5	1
Data repository	Yes	Information repository	5	0
Data warehouse	Yes		157	2
Data mining	Yes		439	1
Online transaction processing (OLTP)	Yes	Online transaction processing	29	0
Online analytical processing (OLAP)	Yes	Online analytical processing	48	0
Expert system	Yes		96	1
Security, privacy and confidentiality of laboratory data				
Confidentiality	Yes		34	0
Security	Yes	Information security	117	1
Health Insurance Portability and Accountability Act (HIPAA)	Yes	Health Insurance Portability and Accountability Act	72	0
Authentication	Yes		45	0
Password	Yes		85	1
Biometrics	Yes		182	3
Audit trail	Yes			2
Encryption/decryption	Yes	Encryption	84	1
Certificate of authority	Yes	Certificate authority	26	1
Firewall	Yes	Firewall (computing)	284	1
SSL	Yes	Transport layer security	179	1
Regulatory issues				
College of American Pathologists (CAP) checklists	No	CAP but no checklists		n/a
American Association of Blood Banks (AABB) accreditation	No	AABB but not accreditation		n/a
Food and Drug Administration (FDA) accreditation	No	Food and Drug Administration (United States) but not accreditation		n/a
Clinical Laboratory Improvement Act (CLIA)	Yes	Clinical Laboratory Improvement Act, 1988	0	0
Quality assurance	Yes		112	1
Quality control	Yes		115	1
Digital imaging and telepathology				
Pixel	Yes		105	0
Resolution	Yes	Image resolution	27	1
Color depth	Yes		60	1
Compression (lossy)	Yes	Lossy compression	36	0
Compression (lossless)	Yes	Lossless data compression	36	1
Compression ratio	Yes	Data compression ratio	10	1
Image analysis: quantitative and qualitative	Yes	Image analysis	8	1
Whole slide scanning	Yes	Virtual microscopy, digital pathology	4, 17	0, 0
Image databases and storage systems	Yes	Image retrieval, picture archiving and communication system	8, 108	0, 0
Emerging technologies				
Voice recognition in computer systems	Yes	Speech recognition	109	3
Laboratory automation systems	Yes	Laboratory automation	5	0
Genomics	Yes		23	0
Proteomics	Yes		24	0
DNA chip arrays	Yes	DNA microarray	82	0
Tissue microarrays	Yes		7	0
Nanotechnology	Yes		530	0

1 The topic heading “Laboratory information system” was included as a term

For articles that mapped to terms, we used the number and type of “tags” or “Template Messages” on each article as a proxy of completeness and/or quality. In Wikipedia, template messages are used for a variety purposes, including indentifying articles that need improvement. Typical reasons for a tag would include problems with reference or citations, redundancy, requests for expansion, addition, background information or major upgrades, disambiguation, copyright issues, etc. (http://en.wikipedia.org/wiki/Wikipedia:Template_messages).

Finally, over a period of 2 days (03/05/2010–03/06/2010), the authors chose a set of 40 articles at random (from the set of articles that mapped to the 199 terms of the 2003 proposal) and evaluated each article on a five-point Likert scale (http://en.wikipedia.org/wiki/Likert_Scale) for completeness (“comprehensive”), quality and lack of major errors (“high-quality”), appropriateness as a beginning text for pathology resident (“appropriate for beginner”), appropriateness as a text for advanced learners (“appropriate for advanced learner”), and its state of being up-to-date (“current”) with the latest knowledge. The editorial activity per year could be quantified using revision history statistics that were also included as a marker for currentness. Finally, the reviewers gave a qualitative (1 = yes, active; 0 = no, non-active) assessment of the activity of discussion and edits being made on the articles.

While the majority of terms in the 2003 curriculum had relevance/interest to pathology informatics and to the wider “IT community” (e.g. “programming language”), there was a subset which was more specific to pathology informatics itself (e.g. “laboratory information system”). Of the 40 articles selected for evaluation discussed in the previous paragraph, 10 were felt to be specific to the “pathology informatics” subset. The Likert scores from the two groups were compared using the Wilcoxon-Mann Whitney test to see if there was a statistically significant difference, with the expectation that the articles of general interest would have higher scores (one-tailed *P* value).

### Organization and Platform

The pathology informatics curriculum wiki is currently hosted by Wikispaces (http://www.wikispaces.com) at http://pathinformatics.wikispaces.com. Wikispaces is used by a number of universities, including Columbia University and University of Massachusetts Lowell, for educational purposes. It is one of many very inexpensive and capable wiki hosting options available, and is very useable and flexible. It should also be noted that the entire contents of the curriculum can be easily and simply moved to another hosting option, if necessary.

The curriculum in the pathology informatics curriculum wiki is based on the previously published standardized pathology informatics curriculum.[34] The content on the curriculum wiki links and organizes Wikipedia articles to a series of educational “chapters” or “modules” on pathology informatics. Each module includes sections for goals, a list of terms/topics (each term/topic is linked to content in the form of Wikipedia articles and other stable online resources), a narrative section (to relate the terms/topics to each other), recommended laboratory-like activities, supplemental online resources for advanced students (*vide* infra), and questions [Figure 2].

**Figure 2a:**
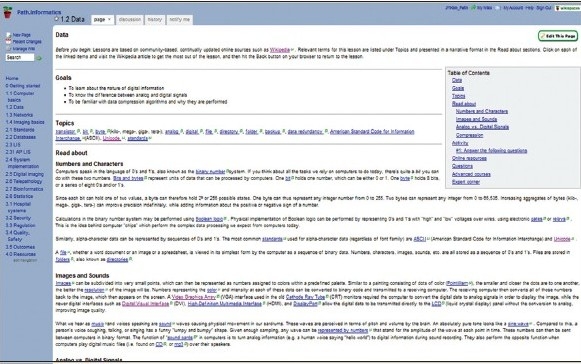
The first half of a screenshot of one of the chapters in the pathology informatics curriculum wiki.

### Improving or Supplementing Wikipedia Articles

Toward the end of the lessons, there are links for “supplemental material” that reference advanced Wikipedia articles or Massachusetts Institute of Technology (MIT) OpenCourseWare (OCW; http://ocw.mit.edu). Through the OCW initiative, MIT makes materials used in MIT courses freely and openly available for noncommercial educational purposes, under their Creative Commons license.[65] Other publicly available, independently well-maintained sites are also referenced, such as the archives of the APIII (http://apiii.upmc.edu).

At the end of each curriculum module, there is an “Expert Corner” where readers can comment on Wikipedia articles. The goal of the “Expert Corner” is to allow readers to identify content that, in the opinion of the reader, needs further improvements (or perhaps issues for which no Wikipedia article exists). Because the core content available on the pathology informatics curriculum wiki resides in community-created and maintained articles in Wikipedia, a user of the curriculum wiki who feels that a Wikipedia article can be improved can, and should, begin a discussion in Wikipedia about changing or extending the article (to see how this is done, go to http://en.wikipedia.org/wiki/Wikipedia:Introduction). However, if the reader does not have the confidence or knowledge needed to improve the article directly, he or she can identify the article for potential improvement by another member of the pathology informatics community. After users of the curriculum highlight or suggest Wikipedia articles relating to pathology informatics, pathology informatics “experts” across the world could easily join Wikipedia and improve, or create, the articles directly (*vide* infra, conclusion).

A potentially important role for the “Expert Corner” is link maintenance. The pathology informatics wiki focuses on stable, core content sources like Wikipedia and MIT, which should minimize the frequency of broken links. Having said that, broken links happen. Ideally, wiki users would fix broken links as they identify them; however, if this is not possible, the problematic links should be at least noted in “Expert Corner” so that others can effect the repair.

### Brief Examination of Rules for Fair Use

The value and content in the pathology informatics curriculum wiki is in the use of links to materials on Wikipedia, MIT OCW and other third-party sites in a way that repurposes the preexisting materials to educate a new audience. Legal issues may arise when linking to third-party sites when the linking page uses copyrighted or trademark-protected text or images from the linked site. However, in this case, Wikipedia operates under the Creative Commons Attribution-ShareAlike 3.0 Unported License which allows users to copy, distribute and transmit its work freely, and its licensing policy requires content hosted on Wikipedia to be free content.[66] Similarly, MIT OCW uses the Creative Commons license to allow non-commercial use of its materials.[67]

At the same time, teaching is an activity that falls under the fair use doctrine of copyright law. Simply stated, the United States and other countries recognize fair use as being “the right to use copyrighted material without permission or payment, when the benefit to society is larger than the damage to the copyright holder”.[68] Because the pathology informatics curriculum wiki is a non-commercial educational website, linking to third-party sites will be included under the terms of fair use. Even so, the majority of the links are to Wikipedia and other websites that are using the Creative Commons license, where this is not an issue.

## RESULTS

### Evaluation of the Pathology Informatics Content in Wikipedia

From the 199 non-duplicative terms published in the 2003 standardized basic pathology informatics curriculum, including the addition of the “Laboratory Information Systems” section header as a term, 179 (90%) have at least one Wikipedia entry and 20 (10%) do not have an entry (as of 03/06/2010; Appendix). The majority of the absent terms (*n* = 15/20, 75%) in Wikipedia were related to components of the LIS [Table 3].

**Table 3:** Summary statistics on pathology informatics terms in Wikipedia

Topic heading	Total terms^1^	Terms found in Wikipedia, *n* (%)	Tagged terms, *n* (% of terms found in Wikipedia)	Average number of tags per tagged term, *n*
Computer basics	20	20 (100)	11 (55)	1.4
Hardware	22	22 (100)	10 (45)	2.7
Software	13	13 (100)	10 (77)	2.0
Computer networks	29	29 (100)	18 (62)	1.6
Internet-related	20	20 (100)	10 (50)	1.2
Laboratory information systems^2^	27	12 (44)	8 (67)	2.0
Data standards and encoding schemes	9	8 (89)	3 (34)	1.0
System management and software development	17	16 (94)	11 (69)	1.5
Data analysis	13	13 (100)	7 (54)	1.1
Security, privacy, and confidentiality of laboratory data	11	11 (100)	8 (73)	1.4
Regulatory issues	6	3 (50)	2 (67)	1.0
Digital imaging and telepathology	9	9 (100)	5 (56)	1.0
Emerging technologies	7	7 (100)	1 (14)	3.0

1 There were four duplicative terms, which were included in the total calculations under each topic heading (total including duplicates, *n* = 203).

2 The topic heading “Laboratory information systems” was included as a term, and was present in Wikipedia. Figures in parenthesis are in percentage

Of the 179 Wikipedia entries mapped to pathology informatics curriculum terms, 102 (57%) entries had one or more “tags” placed on them that highlighted a need for additional improvements to the article. The most common reason for the “tag” was a need for more citations in the article [Table 3].

Twenty-two percent (40 of the 179) of the 2003 curriculum terms that had Wikipedia articles were selected at random and included in a more comprehensive review. The results are shown in Table 4. Among the five qualities assessed on the five-point Likert scale, the Wikipedia articles scored the highest marks (4.18) for being up-to-date (“current”). This correlated with an average of 112 revisions per article over a 1-year period (02/01/2009–02/01/2010). This was followed by “quality” (4.08), “completeness” (4.05) and “appropriate for advanced learners” (3.93). The lowest marks were given in the category of “appropriate for beginners” (3.85).

**Table 4:** Analysis of select terms using a Likert scale for the first five categories

Term	Comprehensive^1^	High-quality^1^	Appropriate for beginner^1^	Appropriate for advanced learner^1^	Current^1^	Number of revisions from 02/01/2009–02/01/2010	Activity level^2^
Computer	5	5	5	5	5	134	1
Bit	5	5	5	5	5	90	1
Directory	5	5	5	5	5	24	1
Unicode	5	5	4	5	5	106	1
Central processing unit (CPU)	5	5	3	5	5	396	1
Redundant array of independent/inexpensive disks (RAIDs)	5	5	4	5	5	398	1
Floppy disk	5	5	4	5	5	262	1
Microcomputer	5	5	5	5	5	57	1
“Dumb” terminal/computer terminal	5	5	5	5	5	18	0
Programming language	5	4	3	5	5	286	1
Local area network (LAN)	5	5	5	5	5	153	1
Gateway	2	3	3	3	3	30	0
Network operating system	3	3	3	2	3	34	0
Print server	4	3	3	3	4	13	0
Thin client	5	5	5	5	5	132	1
Integrated services digital network (ISDN)	5	5	3	5	5	116	1
Internet service provider (ISP)	2	3	3	2	3	88	1
Web browser	5	5	5	4	5	239	1
File transfer protocol (FTP)	4	4	4	4	4	188	1
Markup language and tags	5	5	5	5	5	77	1
Order entry^3^	3	4	4	3	4	39	1
Bar code	5	5	5	5	5	285	1
Query language	3	3	3	2	3	11	1
Health Level 7 (HL7)^3^	5	4	4	5	4	39	1
Logical Observation Identifier Names and Codes (LOINC)^3^	3	4	4	4	3	4	0
Digital Imaging and Communications in Medicine (DICOM)^3^	3	3	4	4	5	76	1
Application service provider (ASP)	2	3	4	3	2	13	1
Technical specifications document	4	4	4	4	4	100	1
User support	4	4	4	3	4	91	1
Object-oriented database	5	4	3	4	4	23	1
Data warehouse	4	4	4	3	5	157	1
Confidentiality^3^	3	3	4	3	3	34	0
Health Insurance Portability and Accountability Act (HIPAA)^3^	4	5	4	5	5	72	1
Biometrics^3^	3	2	2	2	3	182	1
Secure sockets layer (SSL)	5	4	2	2	4	179	1
Quality assurance	2	3	2	2	3	112	1
Compression (lossy)	4	4	4	4	4	36	1
Image databases and storage systems^3^	4	4	5	5	4	8, 108^4^	1
Laboratory automation systems^3^	1	1	1	1	1	5	0
DNA chip arrays^3^	5	5	5	5	5	82	1
Average	4.05	4.08	3.85	3.93	4.18	112	0.83 (active)
Articles of wider interest to the community	4.27^5^	4.27^6^	3.90	4.00	4.33	128^7^	0.87
Articles of specific relevance to pathology informatics^3^	3.40^5^	3.50^6^	3.70	3.70	3.70	64^7^	0.70

1 Likert scale was used for assessment purposes: 1 = strongly disagree, 2 = disagree, 3 = neither agree nor disagree, 4 = agree, 5 = strongly agree;

2 Qualitative assessment score for activity level: 1 = active, 0 = nonactivel;

3 Articles of specific relevance to the pathology informatics community;

4 For calculation purposes, we chose the higher score of 108, which corresponded to one of two possible alternative Wikipedia terms, “Picture archiving and communication system” (versus “Image retrieval”);

5 One-tailed Wilcoxon-Mann Whitney test, *P* = 0.020;

6 One-tailed Wilcoxon-Mann Whitney test, *P* = 0.046;

7 One-tailed Wilcoxon-Mann Whitney test, *P* = 0.043

As can be expected, articles that were of wider interest to the Wikipedia community (e.g. articles of importance to both the general information technology industry and pathology informatics) received higher scores in all categories when compared to articles that could be considered more specific to pathology informatics. The differences reached statistical significance in the categories of completeness (“comprehensive”; *P* = 0.02) and quality (“high-quality”; *P* = 0.046). In addition, the number of revisions per year was significantly higher in the group of articles that were of general interest than the articles specifically relevant to pathology informatics (128 versus 64, *P* = 0.04).

### Organization of the Lessons and of the Curriculum

The current pathology informatics curriculum wiki consists of 20 chapters: Getting started, Computer basics, Data, Networks, Imaging basics, Standards, Databases, LIS (Laboratory Information System), AP LIS (Anatomic Pathology Laboratory Information System), System implementation, Digital imaging, Telepathology, Bioinformatics, Statistics, Hospital systems, Security, Regulation, Quality and Safety, Outcomes, Resources.

An example of a chapter can be seen in Figures 2a and b. Each chapter starts with a template that has sections for: the goals of the lesson, a list of relevant terms and topics that are linked to Wikipedia or other online, stable resources, a narrative section, recommended activities, other supplemental online resources such as didactic materials on MIT OCW, questions, and the Expert Corner where users can indicate Wikipedia articles that need additional help.

**Figure 2b:**
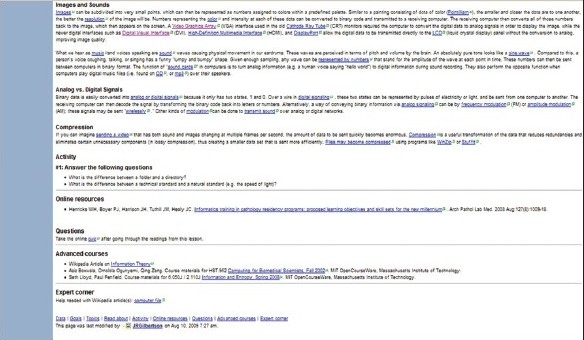
The second half of a screenshot of one of the chapters in the pathology informatics curriculum wiki.

By the nature of the wiki software, users can click on the tabs at the top of each page to join a discussion on that page, look at the history of edits made on the page, or request notification any time a change to the page is made. Every page can be edited by selecting the “Edit this page” button on the top right of the page, and edits may be made anonymously or by a registered user.

## COMMENTS

### Web 2.0, the Pathology Community and the Importance of Pathology Informatics on Wikipedia

For the past 15 years, the internet has provided an increasingly effective medium for the distribution of educational information from a single institution to the rest of the pathology informatics community. With the rise of Web 2.0 technologies, the internet has also become a medium for the distribution and creation of educational information from the community for the community. This is important, as the size, scale and need for informatics training in pathology is ever increasing, and is perhaps beyond what can be readily supported by local initiatives.

Wikis in particular have proven very effective in the collaborative creation and maintenance of knowledge on the web. By their nature, they actively engage teachers and learners in their construction and distribution of knowledge. They are easy to use, and the technology is widely available with open source, free or low-cost software and hosting options.

The information in public, online wikis is immediately available to every pathology informatician with an internet connection, anywhere in the world. Equally important, and more revolutionary, the privilege to create and edit this information is not reserved for specific universities or teaching institutions. This is potentially transformative and useful, as most knowledge in pathology informatics resides outside the academic pathology programs in large and small practices, reference labs, coroner's offices, the LIS, middleware and imaging industry, with engineers, scientists, technicians, histologists and others not normally involved in pathology informatics education. Leveraging the entire community is important as the size, scale and need for informatics education in pathology is larger and is growing faster than academic informaticians can be trained and supported.

Wikipedia, formally launched on 01/15/2001, is the largest wiki and has become the *de facto* first-line encyclopedia for a large percentage of the general population. Although not without controversy or critics, early concerns that large public wikis would be prone to vandalism, unreliability or inaccuracies due to their openness, have not materialized. Although anyone can post content that is misleading, unsuitable or wrong, major wikis have software that tracks versions of a page, allowing an administrator to recover the latest non-vandalized version. A large and active author base combined with the wiki's very openness seems to foster a “socially Darwinian process,” whereby pages are subject to a selection process that favors a middle ground of generally agreed upon knowledge. Sentences and sections can be discussed, edited and replaced if not considered “fit,” which eventually results in a higher quality page. A relatively recent comparison of Wikipedia and the *Encyclopedia Britannica* published in *Nature* showed similar numbers of errors in both the online encyclopedias, indicating that their accuracy may also be similar,[69] though these claims have been disputed by *Encyclopedia Britannica*.[70] A discussion on the reliability of Wikipedia, with references to multiple studies can be found at http://en.wikipedia.org/wiki/Reliability_of_Wikipedia.

Another important concern has been the lack of clear and complete authorship information. Especially for places of higher education, where individuals are traditionally recognized for their efforts by attributed publications, wiki anonymity and collaboration raises a number of issues. The most obvious is the issue of assigning value for the effort required to create and edit content. While the success of Wikipedia is a clear evidence that volunteer, largely anonymous collaboration can produce outstanding work, it would be ideal, and perhaps in some cases necessary, to find a mechanism to recognize effort in creating and sustaining pathology informatics articles (*vide* infra). The wikis themselves could perhaps address this criticism by developing user hit counters and an associated user rating systems of materials read.

### The Strengths and Weaknesses of Wikipedia in Pathology Informatics

The great majority (179 of the 199) of non-duplicative terms in the 2003 API curriculum have at least one related Wikipedia article, and the great majority of these articles seem (in the opinion of the authors of this study) to be comprehensive, of high quality, current and appropriate for both beginners and advanced learners. These results are compelling and support the thesis that Wikipedia articles can be used as the foundation for a curriculum in pathology informatics.

On of the most surprising results of our analysis was the comprehensiveness of Wikipedia articles and their appropriateness for both beginners and advanced learners. In fact, the articles as a group were felt to be slightly more appropriate for advanced learners than for beginners (although the difference was very small and not statistically significant). Thinking this through, however, we should be surprised; Wikipedia articles are created and maintained by volunteers passionate about, interested in and having an expertise on the articles they create. The articles are often long and comprehensive with multiple hyperlinks and citations.

Perhaps the most concerning result in this project to date has been the relative weakness in Wikipedia articles on specific, core topics in pathology informatics. Of the 199 terms from the 2003 curriculum, 20 did not have Wikipedia entries, and of these 75% were related to components of laboratory information systems. To look at this in a slightly different way, slightly more than half of the terms associated with the LIS did not have Wikipedia articles (*n* = 15/27, 56%).

Furthermore, the authors of this study found that even when articles on pathology informatics specific issues such as laboratory information systems, laboratory automation, and laboratory regulatory issues existed, they were significantly weaker (in terms of completeness and quality) than articles on “general informatics and imagingȁ issues such as computers, software, microscopy, digital imaging, etc.

The authors do not find this discrepancy to be a fatal flaw. The quality of articles in Wikipedia is a function of the interest of the Wikipedia audience – it makes sense that articles that involve more people will be better, all else equal, than articles of interest to a smaller group. In fact, we consider this an opportunity: once weak articles are identified though projects like this, it is well within the capacity of the pathology informatics community to address them (*vide* infra).

Identifying (and improving) weak or nonexistent Wikipedia articles important to the teaching of pathology informatics is valuable not only for the teaching itself, but also for the appreciation and understanding of pathology informatics by the general public. Politicians, reporters, venture capitalist, medical students, administrators and our physician colleagues increasingly rely on Wikipedia as the first resource on many medical topics. The Wikipedia community is currently developing “WikiProject Medicine,” a project that “aims to enable Wikipedians to cooperate, organize, make suggestions and share ideas on the improvement of medical and health-related articles of Wikipedia” (http://en.wikipedia.org/wiki/Wikipedia:WikiProject_Medicine). When key components of the pathology informatics infrastructure and knowledge base are not represented in the first-line encyclopedia for an increasing fraction of the population, it bodes poorly for the public understanding of, and decisions about, our field.

### The Value of the Pathology Informatics Curriculum Wiki

If the core content of a pathology informatics curriculum can be found in Wikipedia (and other stable websites), what is the value of the pathology informatics curriculum wiki at http://pathinformatics.wikispaces.com? The goal of the curriculum wiki is to 1) index, organize and present the pathology informatics content (largely in Wikipedia) in the form of understandable and coherent courses, 2) supplement core content with activities and other more advanced content such as the MIT OCW and 3) help highlight areas in which the core content sources (like Wikipedia) need to be created, edited, enhanced or updated.

To this end, the curriculum wiki indexes and organizes Wikipedia articles (associated with the 2003 curriculum) into modules. It puts each term (linked to a Wikipedia article) in context in a short narrative, associates activities and additional resources and, through its “Expert Corner” section, allows readers to highlight Wikipedia content that should be improved or articles that should be included.

Importantly, the online pathology informatics curriculum wiki presented here is intended as a supplement to, not a substitute for, formal departmental support for informatics-related education. Furthermore, training in pathology must go beyond informatics didactics – it needs to involve residents in ongoing departmental activities in information operations, creation, management and communication (e.g. LIS operations, meetings on quality, imaging, electronic medical records, electronic order entry, structured data, reporting critical values, etc.).

### Next Steps

The pathology informatics curriculum wiki is a public wiki that indexes a larger public wiki, Wikipedia, and other web content around the standard pathology informatics curriculum approved by API in 2003. Like all wikis, it seeks to harness user generated content from the entire pathology informatics community to maintain and improve the index, the modules and the content being indexed. Its success will rely heavily upon anonymous volunteerism, and the informal collaboration and vigilance supported by this approach has both strengths and weaknesses.

Given the dynamic nature of pathology informatics (and the very essence of wikis), this project will never be “finished”. The curriculum is 7 years old, not all of the modules have been indexed, not all of the underlying articles are adequate and the curriculum, the index, the articles and the field itself will always be a moving target. It is the dynamic nature of pathology informatics (combined with the limited amount of academic resources) that caused us to consider opening the creation and maintenance of educational content to the entire pathology informatics community through “a public pathology informatics wiki that indexes Wikipedia.”

The Partners Healthcare System Fellowship in Pathology Informatics, which currently includes the Massachusetts General Hospital, the Brigham and Women's Hospital and Northshore Medical Center will continue to develop the wiki as a core part of its fellowship program. However, the ultimate success of the pathology informatics curriculum wiki depends on whether it will be accepted by the pathology informatics community outside of Boston, which means that it must become associated with a more national or international entity, organization, society or conference.

It also needs to deal with the fundamental issues of collaboration, credit and value in academia. When a faculty member, fellow or resident writes a paper or develops a local course, this work is credited in curriculum vitae and becomes real currency when it comes time for promotion or recruitment. Work on a wiki does not currently have such value.

In their 2009 paper “Improving Wikipedia: educational opportunities and professional responsibility,” Callis, Christ and Resasco[71] suggest that volunteering to improve Wikipedia (and by extension, other wikis) may be considered a part of professionalism. On the other hand, in other scientific disciplines (such as physics), it is not uncommon to have tens or hundreds of authors on a paper, especially when communicating results of complex and expensive experiments or initiatives. The pathology informatics curriculum wiki (as opposed to Wikipedia) could be considered as such an initiative. Every department of pathology needs to teach pathology informatics as part of its educational and clinical missions. While the wiki to date has been largely the work of a single person (JYK), 18 others have contributed and their contribution is documented in the wiki's history logs. It may be possible, through future publications, to credit the contributors of the curriculum wiki in a way more consistent with academic practice and value.

## COMPETING INTERESTS

The author(s) declare that they have no competing interests.

## AUTHORS' CONTRIBUTIONS

JYK was responsible for the first draft of this manuscript, the initial design of the pathology informatics curriculum wiki and the implementation of the system. JYK and JRG collaborated in the development of the concept and were actively involved in later versions of the draft. All the authors helped evaluate the system and have reviewed and commented on successive drafts of the manuscript and have provided the first author with approval of the final manuscript.
